# Stability and Rheological Properties of the Novel Silica-Based Organogel—A Drug Carrier with High Solubilization Potential

**DOI:** 10.3390/ma18020266

**Published:** 2025-01-09

**Authors:** Grzegorz Suwiński, Izabela Nowak

**Affiliations:** 1 Department of Applied Chemistry, Faculty of Chemistry, Adam Mickiewicz University, Uniwersytetu Poznańskiego 8, 61-614 Poznań, Poland; 2 Przedsiębiorstwo Farmaceutyczne Farmapol Sp. z o. o., Święty Wojciech 29, 61-749 Poznań, Poland

**Keywords:** silica, organogel, drug delivery carrier, solubilization, cosmetic

## Abstract

Dissolution of a poorly soluble active pharmacological substance in a drug carrier usually requires advanced techniques and production equipment. The use of novel carriers such as microemulsions, vesicles, or nanocarriers might entail various limitations concerning production cost, formulation stability, or active substance capacity. In this paper, we present a novel fumed silica-based organogel as a low-cost, simple preparation drug or cosmetic carrier with interesting rheological properties and high solubilization capacity. The objective of the study was to characterize the utility aspects of the new dermatological base with special emphasis on stability, rheology, and release studies. Various formulations of a silica organogel base with poorly soluble active pharmacological substances such as propolis or ibuprofen were prepared and tested. The studies of thermal stress, enforced syneresis, and long-term stability were performed along with analyses of rheological profiles of alkali-dependent sol–gel transformation and organogel release. The new drug vehicle shows high thermodynamic stability, thixotropic rheology and first-order release profile. Such properties are promising for commercial utility as a dermatologically applied base for poorly soluble substances.

## 1. Introduction

Pharmaceutical industry generates growing demand for original and generic active pharmaceutical ingredients (APIs) designed for useful and competitive drugs to be launched on the market. According to the literature, 40% of currently marketed and 90% of developed APIs are considered poorly soluble [1]. This attribute of substance may affect permeability, bioavailability, and even production method issues of potential drugs. The problem, however, is reduced by a growing number of drug carrier technologies invented particularly for poorly soluble substances. The use of these new technologies is aimed at reaching increased bioavailability, biocompatibility, stability, and release control of a drug with a poorly soluble API. The new technologies based on micronization and precipitation (e.g., lipid nanoparticles, nanocrystals), solubilization (e.g., organogels, microemulsions, self-microemulsifying systems), or encapsulation (e.g., vesicles, microcapsules) have been reported in numerous papers [2,3,4]. All these techniques are based on a homogenous distribution of APIs and their introduction in the smallest possible particle size or in solubilized form. Each of the proposed technologies was characterized by its specific production method, selection of drug excipients for choosing the proper one, API characterization, route of administration, production cost, materials, and needed specific instrumentation [5].

Among all the technological solutions proposed for poorly soluble APIs, the preparation of organogel is considered one of the simplest, usually not requiring advanced equipment to produce. Pluronic Lecithin Organogels (PLOs) and Lecithin Organogels (LOs) with various APIs like polyphenols can be used for drug production industrially or manually by pharmacists [6,7]. The biocompatible PLO and LO bases are also utilized as transdermal systems because of their high drug capacity and stable in vitro release profile [8]. Organogels are semi-solid structures in which organogelator immobilizes organic solvents by creating a crosslinked network. There are several classifications of organogels, based on the types of organogelator and the method of binding. For instance, low molecular-weight organogelators (LMOGs, <1 kDa) and polymeric organogelators (POGs, >2 kDa) can be distinguished. These particles are bound through various interactions: physical, including hydrogen bonding, π-π stacking, Van der Waals forces, and chemical, covalent bonds [9]. This non-complex structure is similar in fluid phase immobilization, transparency, and elastic or viscoelastic properties to hydrogels, such as the popular in pharmacy carbomer gels [10,11]. In contrast to hydrogels, in organogels, the interactions between the crosslinked network and the fluid phase are based on weaker bondings. The phenomenon raises the stability issues resulting from the contraction of the network, observed as a leakage of the fluid phase out of the organogel structure [12]. The process, called syneresis, is a characteristic property of organogels and thus is a main issue in the use of an organogel as a long-term stable drug. Another characteristic property of most organogels is thermal transition of the sol–gel state. The drug stability and favorable method of its production can be assessed based on the transition parameters: Tmelt (organogel melting temperature), Tgel (gelation transition temperature), Tsol (organogel liquefaction temperature), and Tform (organogel formation temperature) [13].

The advanced organogel technologies are expanding rapidly in various applications. However, medicinal use is limited to safe and biocompatible organogelators and solvents. A new interesting way of organogelation was proposed with the use of fumed silica-based organogels. In such organogels, the organogelator forms a network of agglomerated primary silica particles, stabilizing the gel structure. Fumed silica is widely used in oral and dermal drugs. It can create transparent gel with nonpolar solvents such as liquid paraffin as well as with polar organic solvents, like glycols. Thanks to these properties, fumed silica is a potent, biocompatible organogelator [14]. A. Patel with co-workers have proposed an interesting technology for such an organogel by building it up with fumed silica and vegetable oil [15]. They explained the agglomerate-based structure of silica and dependence of organogel properties on the content of fumed silica (2.5–15% *w*/*w*). A more complex formulation with Meloxicam as an API was introduced by T. Osmalek in 2018 [16], who revealed promising rheological properties of a non-Newtonian, pseudoplastic organogel formulated with fumed silica and polar organic solvents.

**Figure 1:**
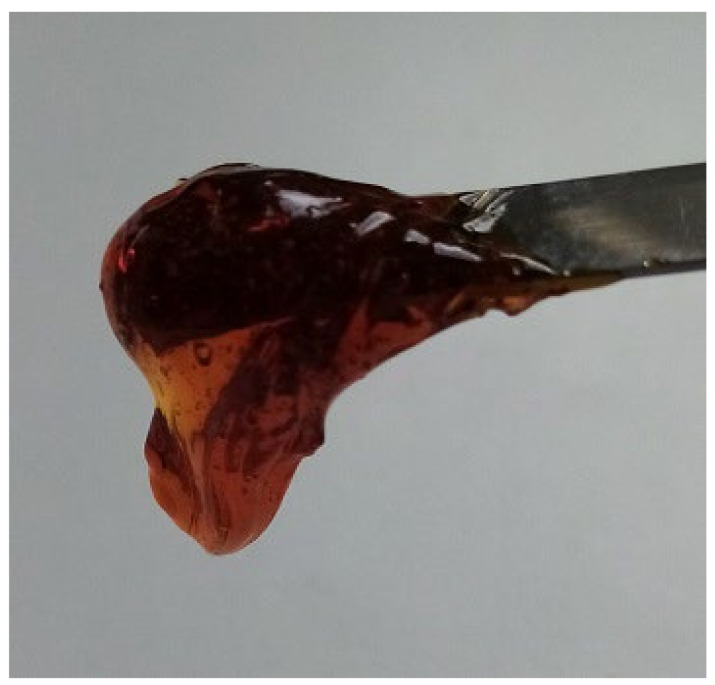
Appearance of propolis organogel.

In this paper, we present for the first time the characterization of novel fumed silica-based organogel formed with a water solution of a base and a weak acid. Recipe foundations for experimental study are described in patent PL 241552 [17]. Experimental methods used in the study were selected for the characterization of utility traits as a drug or a cosmetic with special emphasis on the stability of a silica-based organogel. The vehicle described in this article offers promising advantages over established organogel technologies such as PLO and LO. Similarly, the vehicle can solubilize high contents of APIs (e.g., propolis) and can be produced using non-complex production equipment and non-toxic substances. However, the advantages over PLO and LO may be demonstrated by preferable sensory characteristics (e.g., transparency, Figure 1), similar to those known from popular hydrogels. It also demonstrates a promising prolonged release profile, as evidenced by an example of an ibuprofen-infused organogel.

## 2. Materials and Methods

**Materials**. Fumed silica (Cab-O-sil M5, 200 m^2^/g surface area, Cabot corp., Boston, MA, USA) and propylene glycol were purchased from Brenntag (Essen, Germany). Purified water was acquired from Przedsiębiorstwo Farmaceutyczne Farmapol (Poznań, Poland). Macrogol 400 was purchased from Mosselman (Mons, Belgium). Ethanol 96% (*v*/*v*) was purchased from HGBS (Wrocław, Poland). Glycerin 99.7% (*w*/*w*) was purchased in Oquema (Korschenbroich, Germany). Ibuprofen was kindly donated by Merck (Corsier-sur-Vevey, Switzerland). Propolis was purchased from Standard (Lublin, Poland). All substances used were of cosmetic or pharmaceutical quality. Acids and bases used in the experiment were of analytical quality. Propolis was extracted to liquid propolis extract form by a 2-week extraction of crude propolis closed in cotton filter bags and immersed in a mixture of solvents. The mixture depended on the final formulation described for formulas P2, P6–P8 in Table 1. Propolis extracts were clear liquids with characteristic color and odor.

*Staphylococcus aureus* ATCC 6538, *Escherichia coli* ATCC 8739, and *Pseudomonas aeruginosa* ATCC 9027 were obtained from commercial sources (American Type Culture Collection, Manassas, VA, USA) and stored in Microbank system (Pro-Lab Diagnostics, Round Rock, TX, USA) and inoculated on tryptone soya agar medium. Inoculation was incubated for 24 h at 34.0 ± 1.0 °C. Afterward, one colony was incubated for 16–18 h at 34.0 ± 1.0 °C with liquid tryptone soya medium to obtain a 10^8^ CFU/mL suspension. *Candida albicans* ATCC 10231 was obtained from the Microbank system and inoculated on Sabouraud agar medium. The inoculation was incubated for 48 h at 22.0 ± 1.0 °C. Afterward, one colony was incubated for 48 h at 22.0 ± 1.0 °C in Sabouraud broth medium to obtain a 10^8^ CFU/mL suspension.

**Organogel preparation**. Organogels were prepared according to the patent methodology [17]. Fumed silica was added in portions to organic solvents with solubilized APIs and mixed with a propeller stirrer until a homogenous, viscous liquid was obtained. To initiate sol–gel transformation, a water solution of both basic and acidic substances was added and the contents were stirred until a viscous, semi-solid gel was formed. Propolis organogel was prepared by a different method because propolis extract already contained all the solvents and the water base solution. Propolis extract was placed under a stirrer and silica was added partially until a homogenous, semi-solid organogel was obtained. Xerogel was prepared in the same way as organogels but with slow evaporation of a solvent until dry light mass was obtained. The formulations prepared are listed in Table 1. Formulations 1A-1F were prepared to provide preliminary information on appearance and flow occurrence. Formulations P1–P11 were used for further studies, as described below.

**Methods used for characterization**. Observations were obtained at least in triplicate and their average was taken as the response variable.

*Scanning Electron Microscopy*. The microstructure and morphology of xerogel (**P1**) were determined by scanning electron microscopy (SEM, Quanta FEG 250 (FEI), Hillsboro, OR, USA) under a low-pressure vacuum (70 Pa) and 10 kV beam acceleration voltage. Samples were analyzed on a 400 mesh carbon-coated copper grid.

*Polarized light microscopy*. An even thin layer of propolis organogel (**P2**) was placed on the microscope glass and studied with a polarizing microscope Olympus BX-52 (Tokyo, Japan) with lenses of 10×, 20×, and 50× magnification. Visualization of the sample was performed with signals of height and laser light intensity.

*Particle size distribution*. Volume-weighted particle size distribution was measured on LUMiSizer^®^ 651 (LUM GmbH, Berlin, Germany) according to ISO 13318. Samples (**P3**–**P5**) were homogenized with a spatula and transferred into 10 mm polycarbonate cells. The measurement was performed using 410 nm light, 2300 relative centrifugal acceleration, at 25 °C. The volume-weighted particle size distributions were derived by analyzing the time course of transmission at fixed positions. The particle size was obtained as an equivalent diameter, based on the hydrodynamic diameter of rigid spherical particles.

*Thermal stability of organogel structure*. A sample of 30 g of propolis organogel (**P2**) was placed in a beaker in a water bath set to 70 °C temperature. The sample was submerged in the water bath. After 10 min of heating, the beaker was taken out, wiped off the water outside, and put upside down on a well-lit white background. Any signs of destabilization, liquid separation, or any other visual changes were checked.

*Long term stability*. Propolis organogels (**P2**, **P6**–**P8**), 30 g in polyethylene jars, were divided into groups of test and reference samples. Reference samples were placed in the dark in room conditions at 20–23 °C. The tested samples were placed in a climatic chamber at 30 °C and 60% relative humidity. The tested and reference samples were checked in intervals of 1, 7, 14, 28, 56, 84, 168, and 365 days. Sample inspection concerns changes in the visual appearance of the sample and the container, smell detection, and mass homogeneity evaluation.

*Cooling-heating stability*. Portions of 6 g of propolis organogels (**P2**) in polyethylene small tubes, were divided into groups of test and reference samples. Reference samples were placed in the dark at 20–23 °C. Tested samples were kept in changing conditions: fridge (2–8 °C, 1 day), room temperature (1–3 days), incubator (38–40 °C, 1 day), and room temperature (1–3 days), in a total of 6 cycles. Sample inspection after each cycle concerned changes in the visual appearance of the sample and the container, smell, and mass homogeneity.

**Figure 2:**
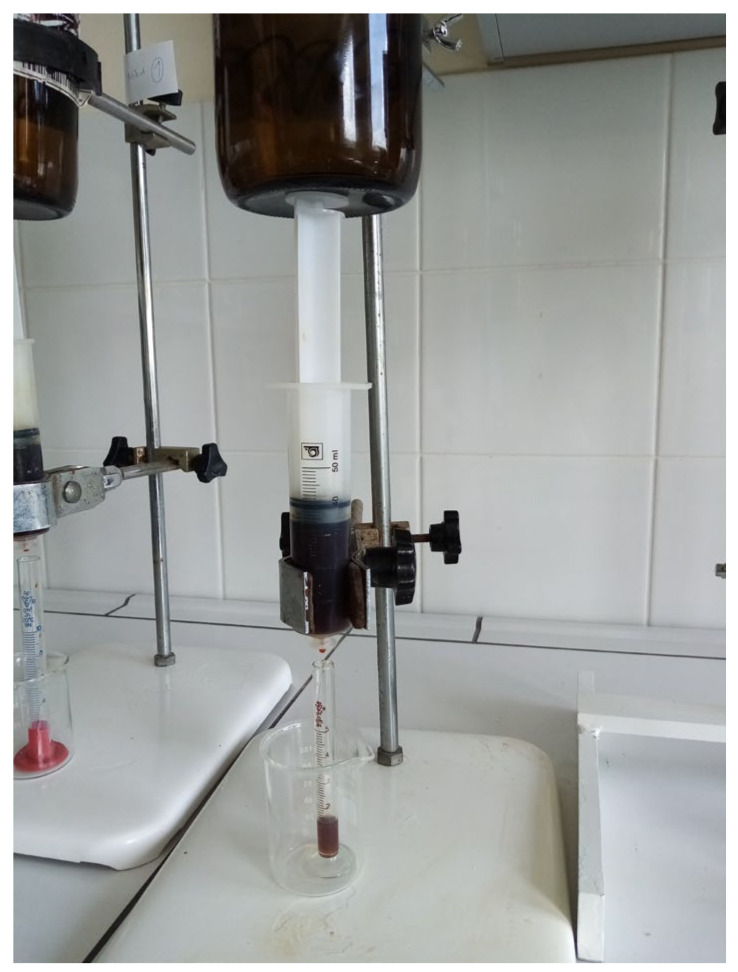
The enforced syneresis measurement system.

***Enforced syneresis***. Enforced syneresis was measured according to the method proposed by Wilhelm and Kind (2014) with modifications [18]. Briefly, a system composed of a sinker (weight), syringe, semi-permeable barrier, and measure cylinder was built (Figure 2). A sample of around 40 mL (V0) of the organogel was inserted into a syringe (Ø = 27.78 ± 0.1 mm) and left for around an hour to stabilize. Then, a syringe piston was inserted and pressed down to full contact with the sample. A constant weight of 1500 g was put vertically on the piston. The volume of the liquid collected (ΔV) in the measuring cylinder was checked at time intervals of 1, 2, 3, 4, 5, 6, and 24 h. The degree of enforced syneresis [%] was measured as equation ΔV/V0. Measurements were performed at 22–25 °C. The samples were prepared using base formulation (**P2**) and different excipients were incorporated into it.

*Rheology profile*. The prepared samples were left for 1 day for structure stabilization. The measurements were performed on an RM180 Mettler Toledo Viscosimeter (Leicester, UK) at 24 ± 1 °C. A concentric cylinder (spindle no. 3) and a 15-step program was used to measure the rheological profile. Shear stress and viscosity were measured after 20 s of each interval from 100 to 500 1/s shear rate. Measurements were made for sol–gel pairs (**P9**–**P10**) to verify the change in the rheological profile before and after the transition.

*Release profile*. The diffusion apparatus 708-DS (Agilent Technologies, Santa Clara, CA, USA), equipped with a UV-Vis Cary 50 Bio spectrophotometer (Varian, Palo Alto, CA, USA, λ = 221 nm) was used for the studies performed under SUPAC-Semisolid Guidance. An Enhancer Cell (VanKeln, Varian, Palo Alto, CA, USA) with a 2 cm^2^ surface area membrane and 200 mL flat bottom vessel was used. A membrane made of a natural polymer that is a special type of cellulose called Cuprophan was used in this study. It is an ultra-thin membrane with a thickness of 7–17 µm and a cutoff value of 7–17 kDa. A major advantage of the Cuprophan membrane is its low risk of perforation due to the high elasticity of the membrane material. The membrane was conditioned in the acceptor solution for several hours before each analysis. The release of ibuprofen from the prepared formulation (**P11**) was studied at 32 ± 0.1 °C using the acceptor solution which was a phosphate buffer of pH 5.8.

**Dermatological study**. The tests were conducted under the supervision of a dermatologist at Specjalistyczne Laboratorium Badawcze Skin Lab INTERNATIONAL Sp. z o.o. with the number of approval of 04/11/19/D/10. Informed and written patient consent prior beginning of tests was collected by the company in accordance with the WMA Declaration of Helsinki and the Product Test Guidelines for the Assessment of Human Skin Compatibility 1997 (Cosmetics Europe) on 15 participants, women aged 18–57. The patch test was performed according to the Jadassohna–Bloch methodology and SCCS notes of guidance for the testing of cosmetic ingredients and their safety evaluation—12th revision. Briefly, a 0.2 g sample of propolis organogel (P2) was placed inside a hypoallergic patch and then onto the participants’ skin on an arm or a back. After 48 h the patch was removed. Verifications of any irritation signs were performed in intervals, 48, 72, 96 h, and a week after the patch application. The visual evaluation was based on the redness level of the skin tone at the site of the patch application.

**Antimicrobial activity**. In tests (compliant with the European Pharmacopeia, briefly in aseptic conditions), 10 g of propolis organogel (**P2**) and reference strains were placed in a flask to obtain a concentration of 10^5^–10^6^ CFU/g of the product, and mixed until homogenous. Portions of 1 mL of the samples were collected after 0 (reference), 0.25, 1, 6, and 24 h. Samples were added directly to broths using a tenfold dilution method with sodium chloride solution and incubated for 24 h at 34.0 ± 1.0 °C for bacterial strains: *Staphylococcus aureus* ATCC 6538, *Escherichia coli* ATCC 8739, and *Pseudomonas aeruginosa* ATCC 9027, and for 48 h at 22.0 ± 1.0 °C for *Candida albicans* strain. Afterwards, the colonies were counted and a weighted average was calculated.

## 3. Results

### 3.1. Observational Study of Organogel Formation

The utility of a given formulation as a carrier for an active substance imposes certain preferred assets, characteristic of hydrogels, e.g., high transparency, not flowing under gravity force, high viscosity, and stability. In this study, various components of the silica organogel were used to distinguish the one showing characteristics closest to commercially popular carbomer hydrogels. The organosol–organogel transition was observed visually by adding an alkaline substance to the solvent with silica and mixing the sample thoroughly with a spatula until gel formation. As observed, alkalis alone induced only labile transition. To ensure permanent gelation, the presence of a small amount of water (2–20% *w*/*w*) and acidic counterions were needed. Formulations **1A**–**1F** are characterized in Table 2. The use of ethanol as a solvent led to the formation of a non-transparent firm gel. The use of glycerin hardly affected the transition but ensured higher viscosity and transparency of the product. The most desirable gelation effect was obtained when using propylene glycol in whose presence a translucent, stable gel was formed.

### 3.2. Microscopy and Structure Confirmation

**Figure 3:**
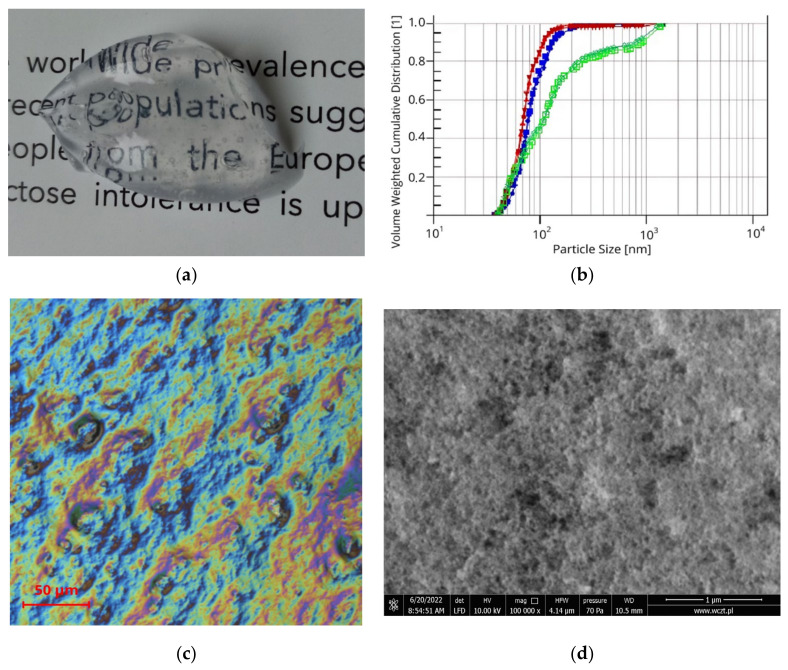
(**a**) Silica-based organogel appearance in macroscale. (**b**) Silica agglomerates particle size distribution: **P3** (red), **P4** (blue), **P5** (green). (**c**) Surface of a propolis organogel (**P2**) in polarized light microscopy. (**d**) Structure of xerogel (**P1**) visualized by scanning electron microscopy.

In macroscale, silica-based organogel appears as a transparent or translucent, homogenous gel. Different microscopic observations were made to check the formation of silica agglomerates (Figure 3). Confocal microscopy with light polarization permitted observations of the irregular surface of the organogel. To obtain SEM images, the organogel was formed and dried into a solid form of xerogel. In high magnitude visualized by SEM, we can distinguish a sponge-like network structure composed of silica aggregates.

This type of structure has different characteristics than linear polymeric structures. For example, a sponge structure with a relatively low solvent–gelator attraction can be the reason for the occurrence of syneresis. An interesting observation is that the size distribution shows almost no enlargement of silica agglomerates between the organosol form (**P3**, median size 70.49 nm), and organogel (**P4**, median size 78.85 nm). The addition of ethanol or other solvents to the organogel affects the size of the agglomerates (**P5**, median size 109.65 nm).

### 3.3. Stability

**Figure 4:**
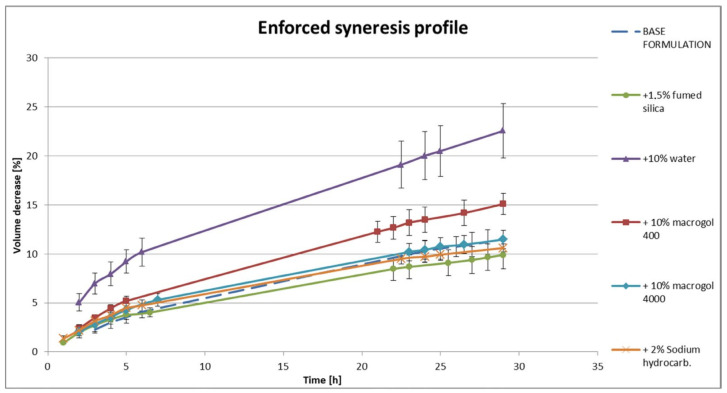
Enforced syneresis alteration by the addition of excipients to the base formulation (P2).

Stability is one of the main features required for a new formulation to be introduced on the market. This is the reason why a couple of methods were used to examine the silica-based organogel stability in the study. So far, a few organogels with incorporated propolis extract have been used in long-term stability studies in a climatic chamber. Most of the tested samples were prone to syneresis and unstable in the long term (**P6**–**P8**). Only one of four tested propolis organogel compositions (**P2**) remained stable for 2 years of storage. It contained both a viscosity-increasing factor (macrogol 400) and a silica agglomeration factor (ethanol), in addition to propylene glycol as a main solvent. However, a high-volume mass storage and confection process may induce an enforced syneresis effect even in potentially stable formulations. To ensure the least possible mass instability, enforced syneresis was measured. Additional excipients, e.g., water, were checked in comparison to the base formulation, and their effect was examined (Figure 4). Some stabilization effect was gained with additional silica or NaHCO_3_ as an alkalic substance. There was a strong effect increasing enforced syneresis after the addition of liquid substances such as water or macrogol 400. To ensure temperature influence on the organogel structure, sample **P2** was tested in a cycle stability study. There was no negative effect on the propolis organogel structure at temperatures ranging from 2 °C to 40 °C. In a short term of 10 min at 70 °C, there was no observed change in organogel appearance.

### 3.4. Rheology

**Figure 5:**
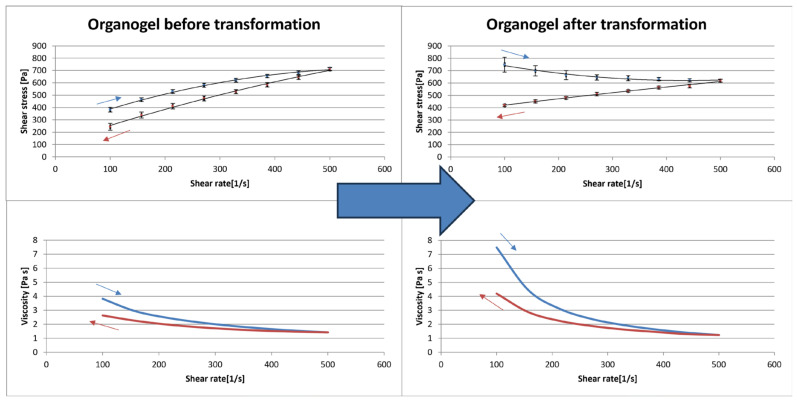
Rheology transformation, including shear stress and viscosity as a function of shear rate, was visualized in paired formulations: **P9** (**left**; before the addition of a basic salt solution) and **P10** (**right**; after the addition of a basic salt solution). Blue coloured dots and line—ascending curve; Red coloured dots and line—descending curve.

Silica-based organogels reveal an interesting rheological shift after the addition of a basic salt solution. This organosol–organogel transition can be observed visually by flow loss under gravity force or via a viscosimeter by determination of rheological profile. The mass of pair **P9**–**P10** formulations before and after the transition reveals non-Newtonian, thixotropic, shear-thinning properties. However, there is an interesting change in the rheological profile ascending curve of organogel. The organosol shows a typical shear stress increase with increasing shear rate, whereas the organogel shows a decrease in shear stress with increasing shear rate. There is also a significant viscosity increase after the transition (Figure 5).

### 3.5. Release Studies

**Figure 6:**
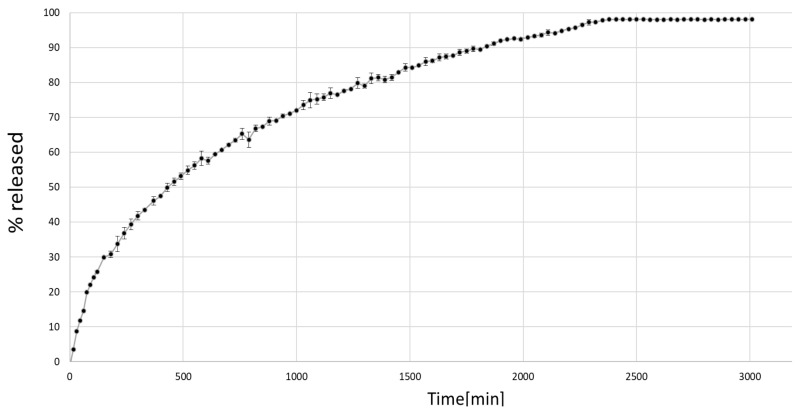
Release profile of ibuprofen from organogel formulation (**P11**).

For the purpose of release studies, the organogel with 10% (*w*/*w*) ibuprofen was tested. Ibuprofen is widely used in dermal applications as a non-steroidal anti-inflammatory drug. Formulation **P11** is an opaque organogel with fully solubilized ibuprofen. Thus, it is an interesting subject for release studies and comparison to ibuprofen in the dermal form of hydrogel. As shown in Figure 6, the organogel containing ibuprofen reveals the first-order kinetics (the natural logarithm of a reactant concentration versus time was linear). It shows prolonged release with over 80% API released in 24 h and full release in 40 h.

### 3.6. Utility Studies

**Figure 7:**
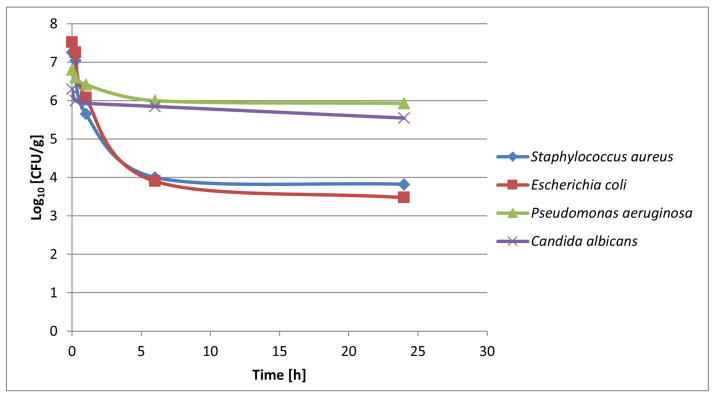
Antimicrobial effect of propolis organogel (**P2**).

The introduction of new technology to the market requires several safety and efficiency studies, dependent on the product registration type and local legislation. Accordingly, a dermatological study (patch test) and antimicrobial study of the propolis organogel were undertaken. The dermal application showed no sign of skin redness reaction after patch removal in all 15 participants. The solvents used in the organogel preparation are widely used and have well-established safety, thus, the organogel composition raises no serious toxicological concern in terms of user safety. The efficiency of the propolis organogel (**P2**) was examined in an antimicrobial study, as this API is usually applied in dermal infections and inflammations. The strongest activity of the propolis organogel was demonstrated against *Escherichia coli* and *Staphylococcus aureus* strains. However, the *Pseudomonas aeruginosa* and mold strain of *Candida* were much less susceptible to the propolis organogel (Figure 7).

## 4. Discussion

Silica gel formation in a pH-dependent manner for water dispersions is a well-examined subject. However, more complex mixtures of aqueous and organic media may affect silica interactions and thus require further investigation; for example, the addition of an alkalic solution to ethylene glycol results in a higher critical shear stress in a pH-dependent manner [19]. According to patent of fumed silica-based organogel, a pH range from 5 to 9 is considered as the best for gel formation with the maintenance of skin-acceptable application [17]. The transition from organosol to organogel in the presence of an alkalic substance and a weak acid salt water solution relies on the silica agglomerates interaction, possibly on electrostatic stabilization. Measurement of organogel zeta potential, however, ended with failure due to methodology limitations. Silica aggregates and agglomerates (>100 nm) in contrast to free silica particles can create a structured network [14]. This type of gel structurization was confirmed by microscopic techniques used in this study. The particle size distribution study brought interesting results proving no effect of the sol–gel transition on the particle size. Such a result may be due to the silica particle diffraction effect and change in the silica attraction forces rather than the growth of silica agglomerates. The permanent gel transition occurs only in the presence of a small amount of water (over 2% *w*/*w*), a base, and a weak acid. This rule includes an API in the role of a weak acid—as presented in the example of propolis. Another important factor for silica agglomerates stabilization is the chemical structure of the solvents present in the formulation. As shown in a simple example of glycerin, propylene glycol, and ethanol, used as solvents, affect the organogel structure in different ways. Ethanol affects firm structurization whereas glycerin affects the lowest silica agglomerates interaction. On the other hand, polyols like glycerin or macrogols, play an important role in increasing viscosity of a composition, which may be crucial to increase its final stability. All the macroscale observed phenomena are based on fumed silica particles surface interactions. Silanol groups in the presence of nonpolar solvents tend to create hydrogen bonds, and in this way, incorporate them into the gelator net structures. However, the organic solvents capable of creating hydrogen bonds disrupt this interaction of silica particles. The viscous character depends on the size and type of silica particles [20]. The gelation in the presented technology is based on electrostatic charge. That is why the addition of a small amount of water is crucial to maintain the charge on the surface. Silanol groups are prone to charge generation in the presence of a strong base. Sodium or potassium counterions enhance silanol dissociation [21]. The increasing pH of silica gels favors the formation of a less consolidated net-like structure [22].

Silica hydrogels and organogels may easily undergo syneresis. The first reason is related to the already mentioned very weak bonding between the gelator and the mixture of the solvents. The second reason is related to the stability of equilibrium between the repulsive and attractive interactions of silica particles. The charge generated on the silica surface can stabilize the net structure but a high pH may determine the formation of siloxane groups from two silanol groups of adjacent silica particles, thus leading to the shrinking effect of the net structure [23,24]. In our study, we proposed two methods for the measurement of syneresis. The first one is the long-term stability study with a visual assessment of the natural syneresis at the time of the product storage. The second one is the enforced syneresis, which is a very important factor for the avoidance of “leakage” during the production process (improper suction force) or storage (high mass weight pressure) and even prediction of natural syneresis with time. Enforced syneresis helps to predict a potential natural syneresis in a much quicker way than prolonged stability studies [18]. That is why it is a very practical method for silica-based organogel formulations. In our study, we confirmed, however, that enforced syneresis cannot disqualify a given mixture as unstable when it is in a small amount. It is a rather comparative method for the prediction of the susceptibility of the tested formulations to natural syneresis. Besides the syneresis control, we were concerned with the thermostability of the silica-based organogel. The specific thermostability distinguishes the silica-based organogel from other types of organogels. The thermal restraint of PEG–EG mixtures influence is worth considering, as the tested **P2** propolis formulation contains a mixture of PEG and PG. M.T. Caccamo and S. Magazù demonstrated that adding EG to PEG increases H-bonded connectivity, resulting in higher resistance of the system to elevated temperatures [25]. Information on thermostability is important in terms of stability and mass packaging in the production process. The cycle stability study showed that lowering storage temperature did not affect the organogel stability.

Rheological study revealed interesting properties of the silica-based organogel and its sol–gel transformation. Viscosity and yield stress increase after the transition is preferable for gel-like drug or cosmetic bases. Occurring transition can be utilized to design proper production technology. Thixotropic properties of the gel state can be helpful in mass packaging as high viscosity is undesirable in this process. Using a stirrer and a piston to exert shear on a dosed mass would decrease its viscosity, thereby making the dosing process more reliable. The rheological study also revealed an atypical shift before and after the gelation. The shear-thinning property of the sol state is connected to the linear arrangement of silica particles, aggregates, and agglomerates after shear stress is increased. This is common for pseudoplastic masses, e.g., creams, pastes, and polymer solutions. However, shear stress reduction in the gel state after the shear rate increase is not common in gel structures. It may be connected to the temporary disruption of silica agglomerates sponge-like network on the surface and the formation of a thin layer of liquid between the spindle and the organogel mass.

The release of ibuprofen from the silica-based organogel was characteristic of first-order kinetics. Compared to various hydrogels with this API, the organogel clearly slowed down the ibuprofen release [26]. This is explained by the good solubility of APIs in the solvent medium, APIs in silica surface adsorption, and the high viscosity of the organogel. However, a high concentration of the active ingredient in combination with prolonged 24 h release may benefit with possible dermal applications. In theory, the organogel solvents may increase permeability as enhancers, but further skin permeability studies are needed.

The utility of the organogel was examined using a substance very difficult to incorporate in a carrier, i.e., propolis. This natural mixture of resins, polyphenols, and waxes is hardly soluble both in water and in an oil phase. For the first time, an almost transparent, high-content propolis organogel in solubilized form was obtained (Figure 7). This substance is widely used in dermatology, urology, and stomatology. For example, propolis extracts are promising for the treatment of acne or herpes virus infections [27,28,29]. As presented in the utility studies, the propolis organogel shows both safety and efficiency in bactericidal activity.

## 5. Conclusions

The design and development of new technologies are very important to numerous branches of industry. Despite the unique qualities of many new technologies, it may be challenging to implement such technologies in commercial production and thus release the product to the market. In our work, we presented a simple, easy-to-produce, and potent carrier for poorly soluble APIs which shows similar characteristics to carbomer hydrogels in terms of transparency, viscoelastic properties, or alkali-dependent sol–gel transformation. The presented example of propolis organogel shows new possibilities for formulating with high doses of dermatological products. Its utility is proven by extended stability, safety, and efficacy studies. The syneresis phenomenon may be of concern, however, it is manageable with proper formulation, production process, and packaging of the product. Moreover, organic solvents used in organogel technology drastically lower the risk of microbial contamination in production. The production process of propolis organogel was established on a production scale and the final product (under the brand of Tisane^®^, propolis gel) was introduced into the market. Fumed silica-based organogels with safe organic solvents may be considered an alternative to the existing organogels and other advanced technologies in terms of industrial production or even as a pharmacy drug, produced manually by pharmacists.

## Figures and Tables

**Table 1:** Formulations prepared in the study.

	Glycerin	Ethanol	Propylene Glycol	Macrogol 400	Water	Fumed Silica	KOH	NaHCO_3_	Sodium Lactate	Propolis	Ibuprofen
1A	83.5	0	0	0	10	6	0	0.5	0	0	0
1B	0	83.5	0	0	10	6	0	0.5	0	0	0
1C	0	0	93.5	0	0	6	0	0.5	0	0	0
1D	0	0	83.5	0	10	6	0	0.5	0	0	0
1E	0	0	83.8	0	10	6	0.2	0	0	0	0
1F	0	0	84	0	10	6	0	0	0	0	0
P1	0	81	0	0	10	8	0	0	1	0	0
P2	0	10	38.3	10	15	8	0.3	0	0	18.4	0
P3	0	0	92	0	0	8	0	0	0	0	0
P4	0	0	87	0	4	8	0	0.4	0	0	0
P5	0	10	61.6	10	10	8	0	0.4	0	0	0
P6	0	0	54.4	20	5.7	10	0.3	0	0	18.4	0
P7	0	12	55.6	0	5.7	8	0.3	0	0	18.4	0
P8	0	0	53.3	10	10	8	0.3	0	0	18.4	0
P9	0	11.1	68.9	11.1	0	8.9	0	0	0	0	0
P10	0	10	62	10	8.5	8	0	0	1.5	0	0
P11	0	10	49	10	10.1	10	0	0.9	0	0	10

**Table 2:** Characteristics of **1A**–**1F** samples. N/A—not applicable.

	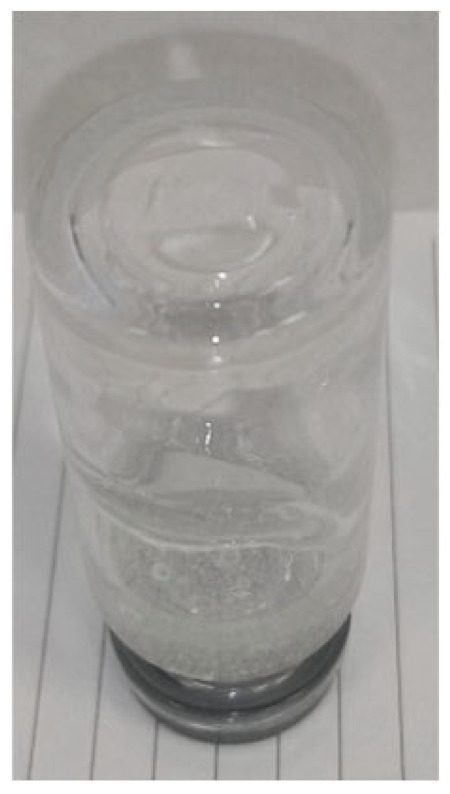	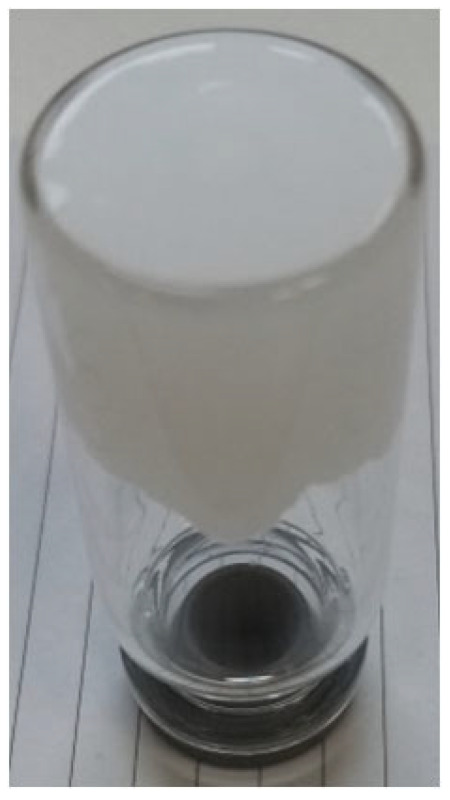	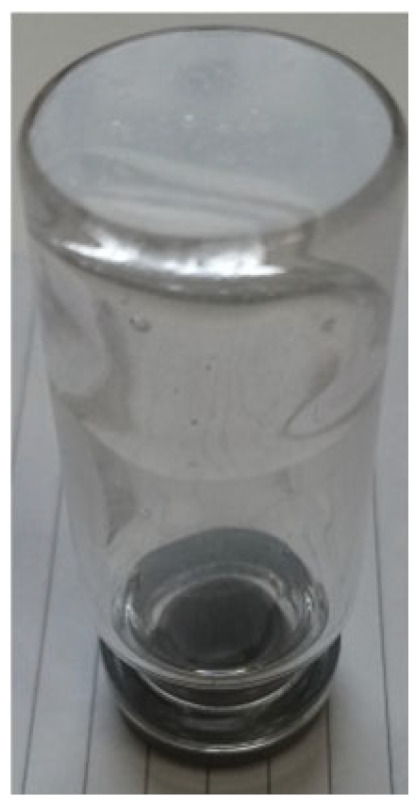	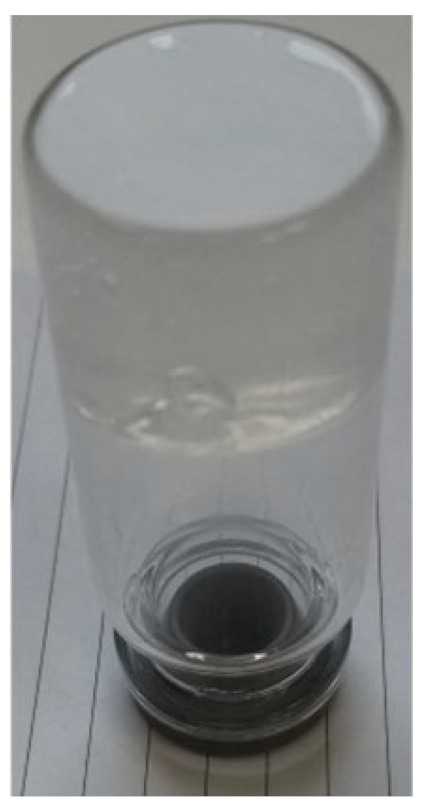	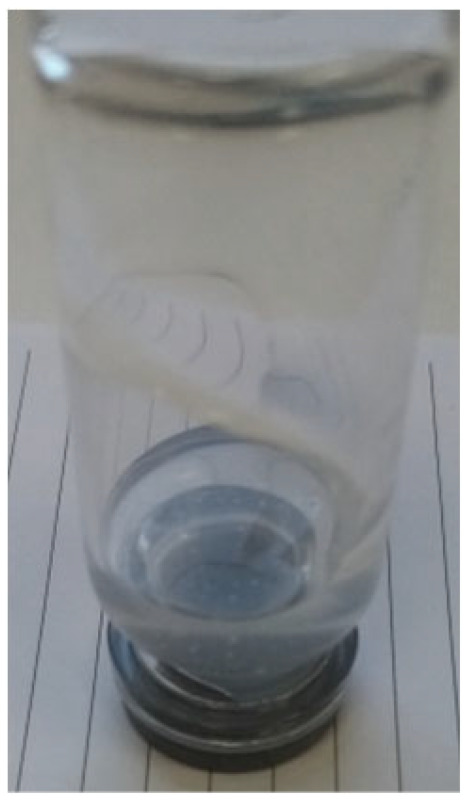	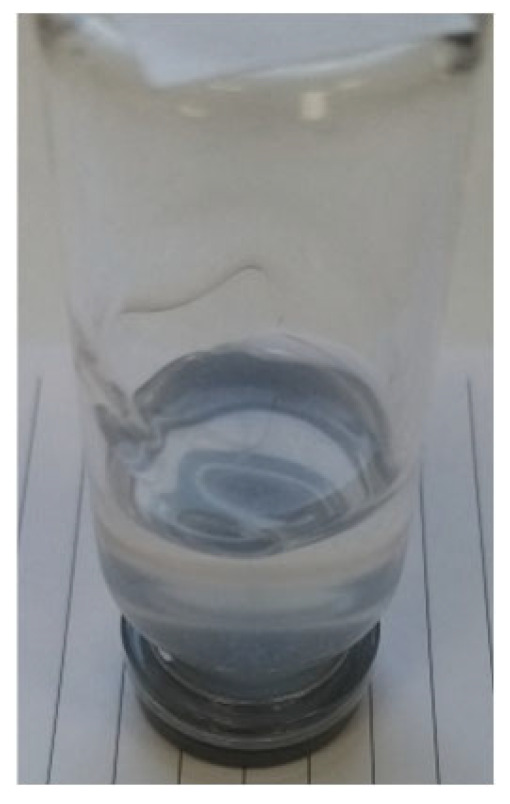
Sample	1A	1B	1C	1D	1E	1F
Flow	YES	NO	YES	NO	YES	YES
Appearance	Transparent	Turbid	Translucent	Translucent	Translucent	Translucent
Syneresis	N/A	YES	YES	NO	YES	N/A

## Data Availability

The data presented in this study are available on request from the corresponding author due to legal.
